# Immunotherapy against lung cancer does not need to compromise the outcomes of COVID‐19

**DOI:** 10.1002/mco2.451

**Published:** 2023-12-29

**Authors:** Jiadi Gan, Jiaxuan Wu, Huohuo Zhang, Dan Liu, Weimin Li

**Affiliations:** ^1^ Department of Respiratory and Critical Care Medicine Institute of Respiratory Health Center of Precision Medicine West China Hospital Sichuan University Chengdu Sichuan Province China

Dear editor,

During the COVID‐19 pandemic, more than 770 million people worldwide have been infected with the causative SARS‐CoV‐2 virus, leading to nearly 7 million deaths.^1^ Since November 11, 2021, the Omicron variant of SARS‐CoV‐2 has received global attention because of its rapid spread, and subvariant EG.5 has become the predominant strain in several areas of the world.^2^ Risk of infection with SARS‐CoV‐2 is higher among individuals with chronic comorbidities such as diabetes, cardiovascular disease,^3^ or lung cancer^4^ than among individuals without such conditions.

Treating COVID‐19 in the background of lung cancer is complicated by the fact that anticancer therapy may adversely affect immune responses to the virus.^5^ Thus, the National Comprehensive Cancer Network recommends delaying immune checkpoint inhibitor therapy in cancer patients with mild or moderate COVID‐19 until at least 10 days after their symptoms have improved.^6^ However, whether such delay is necessary remains controversial. Here, we describe the management of three lung cancer patients who were infected with SARS‐CoV‐2 during the period when the Omicron subvariant BA.5 developed and who showed mild COVID‐19 symptoms (Figure 1A). These cases highlight that immunotherapy, even when started soon after antiviral treatment, can be compatible with good outcomes of mild COVID‐19 in lung cancer patients.

**FIGURE 1:**
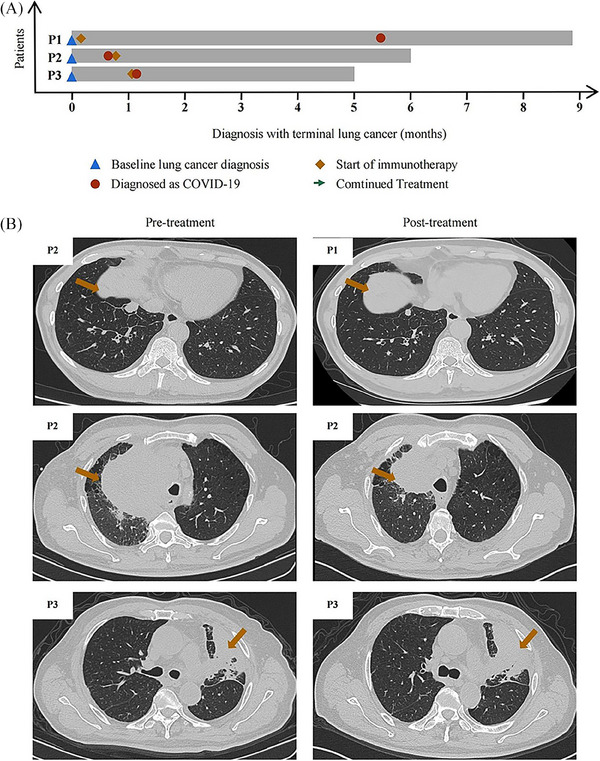
(A) Swimmer's plot of disease course and immunotherapy after COVID‐19 infection in the three patients (P1–P3). (B) Computed tomography of the chest of P1–P3 before and after immunotherapy. Brown arrows indicate pulmonary tumors. P1, patient 1; P2, patient 2; P3, patient 3.

The first patient was a 65‐year‐old Chinese man with a 30‐year history of smoking, who had lung cancer involving a node measuring 1.80 × 1.30 cm in the right lower lung lobe as well as metastases to the liver and bone. Puncture biopsy of the liver lesion indicated a neuroendocrine tumor, while next‐generation sequencing did not detect any known oncogenic mutations. The patient reported fatigue and fever, and nucleic acid testing of a nasopharyngeal swab indicated infection with SARS‐CoV‐2. The patient began to receive first‐line therapy of etoposide, carboplatin, and monoclonal antibody slurry. After 3 months of treatment, computed tomography of the chest showed shrinkage of the lung lesion and the patient was judged to show partial response (Figure 1B).

The second patient was a 63‐year‐old Chinese ex‐smoker in whom enhanced computed tomography of the chest revealed a mass measuring 11.5 × 10.8 cm in the apex of the right lung lobe, as well as bilateral lung and bone metastases. Histology of the lung mass indicated advanced lung squamous carcinoma. The patient tested positive for SARS‐CoV‐2 in a nucleic acid test, but showed no significant COVID‐19 symptoms. He began treatment with pembrolizumab and vibostolimab. After 2 months of treatment, computed tomography showed that the pulmonary lesion had shrunk to 7.1 × 6.2 cm and he was judged to show partial response (Figure 1B). The patient reported feeling better without side effects.

The third patient was a 70‐year‐old Chinese men who reported never having smoked and who had visited another hospital because of chronic coughing, then was admitted to our hospital 3 weeks later because of referral because the persistent coughing did not respond to treatment. Computed tomography of the chest revealed a mass in the left upper lung lobe and left pleural thickening, while magnetic resonance imaging revealed multiple bone metastases but no brain metastasis. Histology of lung lesion biopsy indicated squamous carcinoma, and next‐generation sequencing failed to detect known oncogenic mutations. The patient completed first‐line therapy of four cycles of duvalizumab, albumin paclitaxel, and carboplatin, followed by two cycles of duvalizumab maintenance monotherapy. At 4 weeks after completing these treatments, the patient showed substantial SARS‐CoV‐2 load based on nucleic acid testing of a nasal swab. The patient was given the antivirals nirmatrelvir and ritonavirand for 2 days, after which his coughing improved. He started on duvalizumab maintenance monotherapy. After 1 month, computed tomography showed that the lung lesion had not grown (Figure 1B), and the patient did not report any new symptoms related to SARS‐CoV‐2 infection.

At their most recent follow‐up, none of the three patients reported any symptoms related to SARS‐CoV‐2 infection and they continued to receive anticancer treatment (Figure 1A). Our case series suggests that immunotherapy against lung cancer need not compromise outcomes of COVID‐19, which is an important central contribution by this study. However, larger studies should verify our experience and explore whether it holds true for other Omicron variants and other treatment. Individualized treatment, guided by a multidisciplinary approach, remains critical for optimizing outcomes for cancer patients during the COVID‐19 pandemic.

## AUTHOR CONTRIBUTIONS

Weimin Li and Dan Liu designed the study. Jiaxuan Wu collected the clinical data. Huohuo Zhang analyzed the data. Jiadi Gan created the figure and wrote the manuscript, which all authors revised and approved.

## CONFLICT OF INTEREST STATEMENT

The authors declare no conflict of interest.

## FUNDING INFORMATION

This work was supported by the Clinical Research Incubation Project of West China Hospital of Sichuan University (2018HXFH012), Science and Technology Foundation of Sichuan Province, China (2020YFS0572) and the National Natural Science Foundation of China (82200078).

## ETHICS STATEMENT

No specific approval was needed for this study, since patients were treated during normal hospital operations. All three patients provided written informed consent for their anonymized data to be analyzed and published for research purposes.

## Data Availability

The data in this study are available upon request from the corresponding author and in accordance with local and international rules to protect patient anonymity.
